# Non-coding RNAs as emerging regulators of epithelial to mesenchymal transition in non-small cell lung cancer

**DOI:** 10.18632/oncotarget.16375

**Published:** 2017-03-18

**Authors:** Ying Chen, Lu Lu, Bing Feng, Siqi Han, Shiyun Cui, Xiaoyuan Chu, Longbang Chen, Rui Wang

**Affiliations:** ^1^ Department of Medical Oncology, Jinling Hospital, School of Medicine, Nanjing University, Nanjing, Jiangsu, PR China

**Keywords:** non-coding RNA, non-small cell lung cancer, microRNA, long-coding RNA, epithelial-mesenchymal transition

## Abstract

Non-small cell lung cancer (NSCLC) remains a major health problem that patients suffer from around the world. The epithelial to mesenchymal transition (EMT) has attractive roles in increasing malignant potential and reducing sensitivity to conventional therapeutics in NSCLC cells. Meanwhile, it is now evident that non-coding RNAs (ncRNAs), primarily microRNAs and long non-coding RNAs contribute to tumorigenesis partially via regulating EMT. This article briefly summarizes current researches about EMT-related ncRNAs in NSCLC and discusses their crucial roles in the complex regulatory network. Also, the authors will show the evidence that ncRNAs not only contribute to cancer cells migration and invasion, but also take charge of the resistance of chemotherapy, radiotherapy and EGFR-TIKs. Then, we will further discuss the potential of inhibition of EMT via manipulating relevant ncRNAs to change our current treatment of NSCLC patients.

## INTRODUCTION

NSCLC remains an emerging worldwide health problem, accounting for 85% of the reported cases of lung cancer. Pathological features classify it in three main categories: adenocarcinoma, squamous cell carcinoma and large-cell carcinoma [1–3]. The outcome of patients with NSCLC is poor for nearly half of them with metastatic disease when first diagnosis [4]. Nowadays, patients with advanced NSCLC harboring sensitizing mutations can achieve survival rates at least double by treated with epidermal growth factor receptor tyrosine kinase inhibitors (EGFR TKIs). EGFR TIKs show dramatic tumor response and favorable clinical outcomes *via* competitively inhibiting the tyrosine kinase domain of EGFR [5]. Furthermore, all guidelines recommended that chemotherapy is the mainstay of treatment in NSCLC and adjuvant radiotherapy is commonly useful for patients with limited disease [6]. However, primary and acquired resistance to these therapies remains a major clinical problem.

## NON-CODING RNAs OPEN NEW AVENUES INTO THE BIOLOGY OF NSCLC

The advent of advanced techniques has uncovered that protein-coding genes only represent < 2% of the total human genome sequence. Importantly, these technologies also have revealed the widespread expression of ncRNAs [7]. Currently, this novel class of RNAs are well recognized as versatile and effective regulatory molecules in a wide range of biological progresses [8]. Based on size, ncRNAs can be divided into two classes: small ncRNAs less than 200 bps and long ncRNAs (lncRNAs) greater than 200 bps.

MicroRNAs (miRNAs), with size from 19 to 25 nucleotides, are the best characterized family of small ncRNAs [9]. Increasing evidence has shown that miRNAs play important roles in various biological processes through regulating the expression of genes. About 60% of messenger RNAs (mRNAs) contain target sites of miRNAs, and following polymerase II-mediated transcription, miRNAs bind to mRNAs to mediate gene expression at the post-transcriptional level [10]. MiRNAs are suggested to be implicated with cancer initiation, progression and metastasis due to their frequent deregulation in various types of human cancers, including NSCLC [11]. For example, Let-7 is the first identified miRNA in NSCLC [12]. The let-7 family was later shown to inhibit cancer progression *via* downregulating the tumor promoting protein, such as RAS, MYC, and HMGA2. To date, a number of miRNAs are reported to have a similar tumor-suppressing action, including miR-138, miR-136 and miR-221. In contrast, other miRNAs (miR-21, miR-137 and miR-182-3p, etc) are reported to participate in promoting the development and progression of NSCLC [9]. Taking this into account, the vital field of miRNAs provides a reservoir of new biomarkers for NSCLC. Recently, most studies on the roles of miRNAs have focused on their involvement in chemo- or radioresistance of tumor cells [13]. For example, it was reported that let-7c not only has a suppressor activity, but also reverses chemo- or radioresistance of lung adenocarcinoma cells [14].

On the other hand, lncRNA is the least characterized class, but emerged as a versatile regulator of pathophysiological key pathways. To carry on its functions, lncRNA regulates gene expression through acting on the epigenetic, transcriptional, and posttranscriptional levels [15]. Similar to miRNAs, lncRNAs are gaining increasing attention in the field of NSCLC research. Although dozens of lncRNAs are identified to contribute the development and progression cancer, only few validated lncRNAs have been reported to change expression in NSCLC today [16]. High expression level of metastasis-associated lung adenocarcinoma transcript 1 (MALAT1) was observed to be correlated with poor prognostic in patients suffering from NSCLC. Schmidt et al. has first demonstrated that MALAT1 can mediate metastasis development [17]. Other well-characterized lncRNAs contain HOTAIR, ANRIL and H19, whose expression level also increased in NSCLC, whereas GAS5 and MEG3 are associated with tumor-suppressive function [18–22].

As one of the epithelial tumors, NSCLC recapitulates multiple development programs that promote the occurrence of metastases, including EMT [23]. Recent studies have demonstrated that post-transcriptional regulatory networks, such as miRNAs and lncRNAs, mediate the progresses of EMT.

## MOLECULAR BASIS OF EMT IN NSCLC

During the past 20 years, it has gained more and more attention that the activation of tumor invasion is induced by a phenotype change that recapitulates the EMT event, which not only plays a crucial role in the development of embryo [24]. Increasing evidence has pointed to the role of EMT by which tumor cells would weaken E-cadherin-dependent cell-cell junctions and enhance motility. Recently, loss of epithelial markers such as E-cadherin has been reported to be associated with poor survival in several carcinomas including NSCLC.

EMT is known as a series of events where epithelial cells convert into mesenchymal cells. Invasion causes cancer metastasis, and extracellular matrix (ECM) is a key player in the activities that cause cell translocation into the stroma [25]. ECM, surrounding mesenchymal cells in interstitial spaces, is a source of growth factors, including fibroblast growth factors (FGF), epidermal growth factor (EGF) and hepatocyte growth factor (HGF). In most studied systems, growth factors and their cell surface receptors seem to be implicated as signals for triggering EMT, and ECM-related molecules generate cascades of intracellular signals that converge on the downregulation of E-cadherin. Therefore, the cadherin/catenin/cytoskeleton complex is perturbed, leading to the loss of cell-cell adhesion and reorganisation of the actin cytoskeleton [26]. Finally, the basal surface emanates a cytoplasmic protrusion, allowing cell body detachment from its neighbouring cells. The tumor cell will take on the typical properties of mesenchymal cell, which finally migrate in the adjacent tissue [27].

**Figure 1:**
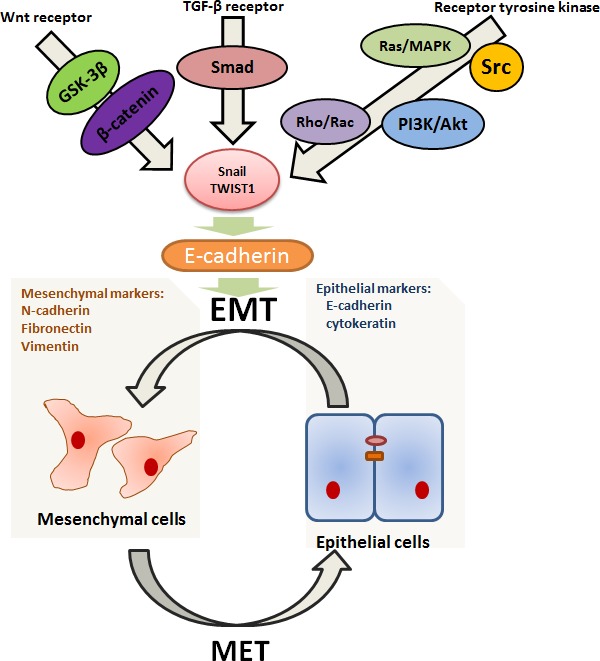
Highly simplified diagram illustrating some better elaborated transduction pathways related to EMT/MET EMT is a highly complex process under the strict control of growth factors and downstream transcription factors. It seems to occur in a context where main roles are played by tyrpsine kinase receptors, Wnt and TGF-β pathways. These pathways are involved in the upregulation of Snail and Twist family *via* signaling through downstream transcriptional factors (such as Ras, PI3K, Rac, Src, Smad and β-catenin), which then represses E-cadherin. What's more, the inhibition of E-cadherin can induce EMT and, on the other hand, its re-activation can stimulate the reverse process, suggesting that E-cadherin takes center stage in mediating the switches between EMT and MET.

E-cadherin is thought to be a crucial component of the cellular signaling network, and the loss of its expression is a fundamental event in EMT [28] (Figure 1). Inaction of E-cadherin play an important role in tumor progression, and action of inappropriate tumor stroma components can also induce EMT in lung cancer. According to the effects on E-cadherin promoter, *the* transcription *factors* can be classified into two groups. Factors, such as Snail, Zeb, E47 and KLF8 repress the activity of E-cadherin by binding to them, and another group is consisted of Twist, Goosecoid, E2.2 and FoxC2, which repress E-cadherin expression directly [29]. In stage I NSCLC, abnormal expression of Twist, Slug and Foxc2 is a significant predictor of overall survival [30]. Knockdown of Snail or Twist, zinc finger transcription factors and key E-cadherin repressor molecules can lead to the restoration of chemosensitivity during EMT [31, 32]. Central in E-cadherin repression is the transcription factor Snail, that is responsible for the disruption of E-cadherin mediated cell-cell contacts and invasion [33]. Snail family is the zinc finger protein, containing SNAI1 and SNAI2 (also known as Slug), that represses E-cadherin transcription through binding the E-box E-pal element in developmental EMT. Different signaling pathways triggering EMT have been found to be linked with the induction of Snail family members [34]. Cigarette smoke extract (CSE) can decrease the expression of E-cadherin through upregulation of Slug and LEF-1 in lung cancer cell lines, demonstrating that cigarette smoking is significantly associated with inducing EMT [35, 36]. Meanwhile, some of these oncogenic pathways regulate Snail by modulating GSK-3β activity. Thus, Snail cooperates with GSK-3β to mediate many signaling pathways that lead to EMT [37].

In most model systems, EMT takes place in a context where growth factors and receptor tyrosine kinases play a main positive role. Upon growth factors binding, receptor tyrosine kinases initiate a series of cytoplasmic transduction events such as activation of Ras/MAPK, PI3K/Akt, Rho/Rac and Src pathways [26]. Ras is a member of small GTP-binding protein that mediates its effects by activating the serine-threonine kinase Raf, which then activate mitogen-activated protein kinase (MAPK) leading directly to Snail family upregulation [38]. In addition to activating the MAPK cascade, Ras can also induce EMT by triggering other effectors such as the phosphatidylinositol-3-kinase (PI3K) and Rho/Rac signaling [39, 40]. PI3K is a lipid kinase which generates phosphatidylinositol-3, 4, 5 triphosphate (PIP3), and in turn, targets the serine-threonine kinase Akt, causing its translocation at the inner cell membrane. In addition to up-regulating Snail, PI3K-Akt signaling is required for TGF-β-mediated EMT [40]. Growth factor-mediated stimulation of receptor tyrosine kinases and their downstream mediators Ras and PI3K, all able can lead to activation of Rho family GTPases. The small GTPase Rho and Ras are involved in cell motility, and the balance between Rho and Rac activities seems to be one of major switch of EMT [27]. Src behaves as a potent trigger of EMT by activating focal adhesion kinase (FAK) directly, which results in activation of the MEK-MAPK signaling [41]. In addition, Src activation induces β-catenin phosphorylation at tyrosine residues, and leads to its binding to E-cadherin, favouring cell dissociation and EMT [42]. Repeated exposure to gefitinib or erlotinib can drive tumor cells into mesenchymal cells. Then cellular dependence on EGFR signaling is suppressed, suggesting that acquired clinical resistance to EGFR-TIKs is associated with EMT [43]. Lung adenocarcinomas harboring EGFR mutations have been shown to exhibit losing expression of IL-6, which is a major activator of the JAK/STAT3 and PI3K/AKT pathways. Based on these results, Li et al. has proved that suppress the IL-6/STAT3 pathway can cause EMT reversal and re-sensitize EGFR-TKI-resistant human lung cancer cells to erlotinib or gefitinib [44]. The NSCLC tumors insensitive to EGFR TKIs include increased protein expression of vimentin, combined with the loss of E-cadherin, claudin 4, and claudin 7 by immunoblotting. Two zinc-finger E-box-binding homeobox factors, ZEB1 and ZEB2, contain a conserved central homeobox region and two zinc-finger domains, and function as inhibitors of E-cadherin during EMT. Indeed, an inverse relationship between E-cadherin and ZEB1 expression has been observed in gefitinib-resistant NSCLC cell lines [45]. Cigarette smoking is also demonstrated to stimulate the EGFR-TKI resistance *via* inhibiting Src activation in NSCLC. And the Src family functions as a regulator of ERK and AKT pathway, which are important signal pathways mediating EMT [41, 46, 47].

Wnts are interesting regulators in EMT and the central component of their signaling pathway is β-catenin. Paradoxically, loss of β-catenin is associated with worsened prognosis in NSCLC, possibly because of its interactions with E-cadherin [48]. This signaling is initiated when Wnt ligand binds to Frizzled proteins, and therefore GSK-3β is inhibited, finally leading to cytoplasmic accumulation of β-catenin. Then, β-catenin functions as a transcription cofactor with TCF/LEF to activate target gene in EMT. Meanwhile, a variety of growth factors or receptors, including E-cadherin, ILK, PI3K/Akt and Ras/MAPK pathways, are intersect with Wnt/β-catenin signaling, and this can enhance their influence on EMT induction [49].

Meanwhile, TGF-β is another potent inducer of EMT. The TGF-β signalling pathway acts through TGF-β type I and II transmembrane serine-threonine kinase receptors to phosphorylate the cytoplasmic Smad2 and Smad3, thus resulting in the activation of the Snail family [50]. Moreover, synergism between IL-6/JAK/STAT3 and TGF-β/Smad signaling, as well as TGF-β and Raf/MAPK, is required to induce EMT in lung carcinomas, suggesting that EMT is orchestrated by several signaling pathways [51]. In addition, it shows that collagen type I can stimulate TGF-β to promote the development of EMT through PI3K-extracellular signal-regulated kinase pathway [52].

Furthermore, lung cancer cells with TGF-β1-induced EMT can acquire stem cell properties, and then exhibit therapeutic resistance [53]. Cancer stem cell (CSC) is a rare subset with the capacity for self-renewal and differentiation with tumors. Emerging evidence supported that lung CSCs is involved in metastasis, as well as resistance to radiation and chemotherapy [54]. TGF-β1-induced EMT in lung cancer cells can drive mobile CSCs from stationary CSCs through the acquisition of a mesenchymal profile which is related to increased levels of stem cell markers [55]. Meanwhile, several signaling pathways, including Wnt, Notch and Hedgehog that mediate EMT, also drive CSCs self-renewal and maintenance [56]. The special properties of CSCs is significantly involved in drug-resistance, such as their high capacity for DNA repair, and high expression of various drug resistance membrane transporters [57].

Interestingly, EMT is not irreversible and the reverse process is mesenchymal-epithelial transition (MET). At the site of metastases, the mesenchymal tumor cells must undergo the reverse transition after the occurrences of EMT, which is critical for distant colonization [23]. Indeed, the alternation between EMT and MET is ongoing between tumor progression [58]. Similar to EMT, MET is also mediated by various effectors. For example, the re-activation of E-cadherin can result in MET [23].

## MICRORNAS-RELATED EMT IN NSCLC

### MicroRNAs function as suppressors of EMT

**Figure 2:**
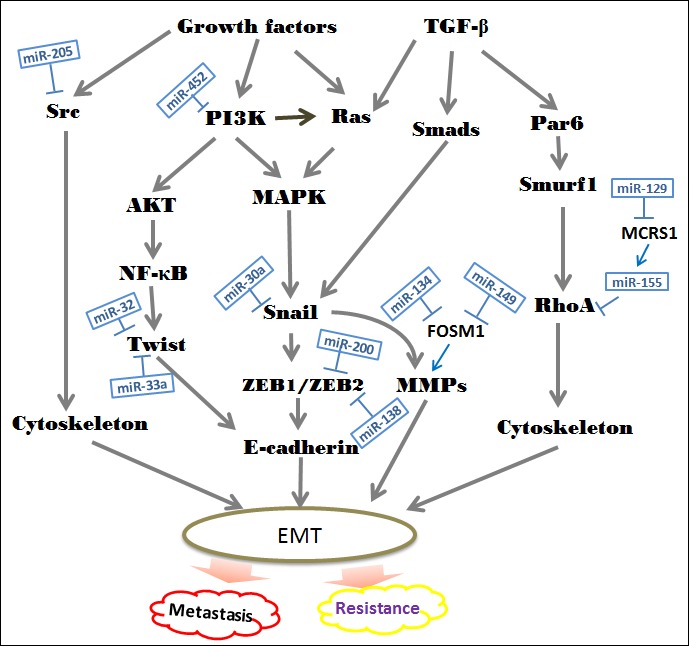
Suppressive roles of special miRNAs in the EMT-associated regulatory networks The diagram shows the major signal transduction pathways leading to EMT in NSCLC. Currently, dozens of miRNAs are identified to function as vital inhibitors in the development of EMT. In NSCLC, loss of these specific miRNAs leads to expression of target oncogenes, which in turn contributes to cancer cell invasion and resistance to chemotherapy, radiotherapy and EGFR-TKIs.

MiRNAs are associated with almost all basic signaling pathways, which make them to be one of most relevant determination of cancer biology. As the list of these miRNAs keeps increasing, the molecular knowledge of miRNAs related to EMT is now considered to be an important focus for NSCLC research [56]. Increasing evidence indicates that specific miRNAs function as powerful suppressors of EMT, such as miR-124, miR-135a, miR-148a and miR-193a-3p/5p, which are essential in tumor cell invasion. Their expression is often downregulated in lung cancer [59–62] (Figure 2).

The miR-200 family, represent by miR-200a, -200b, -200c, -141 and -429, is involved in the regulation of EMT/MET [63]. The EMT event allows the tumor cell to migrate away from the original tissue, and MET enables it to colonize and produce metastases in distant organs. Namely, both downregulation and upregulation of miR-200 family members have been reported to correlate with worse prognosis of patients. Early studies have reported that low levels of miR-200a, miR-200b and miR-429 are correlated with short overall survival in NSCLC patients. In contrast, high miR-141 and miR-200c function as poor prognostic makers [64]. In general, the expression of miR-200 is lost in mesenchymal-like tumor cells and negatively correlated with NCSLC metastasis. Restoring its expression inhibits ZEB1 and ZEB2 leading to the re-expression of E-cadherin [65]. Then, re-activation of E-cadherin will make cancerous cell to loss aggressive, invasive phenotype [66]. Re-expression of miR-200 also drives the significant downregulation of ECM proteins, including collagens and matrix stiffening proteins. These ECM products may function as an effector of TGF-βto induce EMT [67]. Although these miRNAs belong to the same family, they differ in their targets. Hereditary hemochromatosis (HFE) is a predicate target of miR-200a, miR-200b and miR-200c. Inhibition of either HFE or its upstream beta-2-microglobulin (B2M) can reverse EMT [66]. Moreover, recent findings have suggested that miR-200c affects the invasion suppression at least in part through the induction of USP25, which is a member of ubiquitinating-specific proteases family. EMT is intimately interwoven with the ubiquitin proteasome system to regulate the process related to carcinogenesis [68]. ZEB2 is one of the targets of miR-200c and miR-132 that suppress EMT *via* upregulating E-cadherin, downregulating N-cadherin and vimentin expression [69]. In addition to the miR-200 family, miR-489 and miR-638 also have been reported to induce EMT *via* decreasing epithelial makers level and increasing mesenchymal makers levels [70, 71]. FOXM1 is a member of forkhead box (FOX) transcription factors, and participates in early steps of metastasis *via* inducing MMP-2, MMP-9 and VEGF. Through binding to FOXM1 directly, both miR-149 and miR-134 can activate the TGF-β-induced EMT in NSCLS cells [72].

Microspherule protein 1 (MCRS1) is identified as a vital regulator in gene transcription, and observed to promote EMT and metastasis in NSCLC cells. It is directly and negatively mediated by miR-129. MiR-155 may be a functional downstream effector of MCRS1 and regulates TGF-βinduced EMT by targeting RhoA. The miR-129/MCRS1/miR-155 axis seems to have a potential role in mediating EMT-induced metastasis [73]. It is well recognized that the PI3K/AKT pathway contributes to the regulation of EMT. MiR-452 inhibits NSCLC proliferation and metastasis through blocking the activation of PI3K/AKT pathway [74]. TWIST is a key promoter of E-cadherin in NSCLC. Both miR-32 and miR-33a are reported to target TWIST1 directly [75, 76]. Snail, another E-cadherin promoter, was also proved to function as a direct target for specific miRNAs. MiRNA-30a inhibits EMT *via* regulating Snail in NSCLC cells [77]. Additionally, suppression of Let-7 is consistent with the increased expression of the HMGA2, upregulating the TGF-β co-receptor TGFBR3 and thereby promoting EMT traits [78].

In the context, we show the emerging roles of EMT in inducing resistance of lung cancer cells to conventional chemotherapy and radiotherapy, and recent studies also have shown that miRNAs is significantly associated with the regulation of resistance. For example, miR-138 has been demonstrated to sensitize NSCLC cells to ADM *via* directly target ZEB2 [79]. Besides, miR-200/ZEB axis is also responsible for sensitivity to nintedanib in NSCLC cells [80]. The activation of ZEB1 and Src related to induction of EMT is in part mediated by Human CRIPTO1, which is also known as teratocarcinoma-derived growth factor 1 (TDGF1). Overexpression of miR-205 inhibits CRIPTO1-dependent ZEB1, as well as the activation of Src, leading to the restoration of erlotinib sensitivity [81].

The hedgehog (Hh) signaling pathway is a vital mediator of embryogenesis especially in the lung tissue. Activation of the Hh pathway exists in many cancers including NSCLC, which also establishes a mechanistic role in EMT-associated drug resistance phenotype. Re-expression of miR-200 and let-7 family, especially miR-220b and let-7c, causes a direct inhibition of Hh signaling, and thereby diminishes the erlotinib and cisplatin resistance of A549M cells [82]. Meanwhile, miR-200c reversals EMT and then radiosensitizes A549 cells *via* targeting VEGF-VEGFR2 pathway partly [83]. Through cross-talking with several transcriptional factors, Notch signaling pathway promotes EMT, and then affects the sensitivity of cancer cells to treatment in NSCLC. Rhamnetin and cirsiliol can act as novel radiosensitizers *via* increasing the expression of tumor-suppressive microRNA, miR-34a, which leads to the inhibition of Notch-1 expression [84]. Besides, Over-expression of miR-17 can decrease cisplatin-resistant *via* inhibition of TGF-β signal pathway partly. It directly targets and represses TGF-beta receptor 2 (TGF-βR2), and the latter is an important component of TGF-β signal pathway [85].

### MicroRNAs function as promoters of EMT

Breakdown of tight junctions is one of the defining functional changes occurring in the EMT progress, and it is associated with the loss of epithelial markers and acquisition of mesenchymal makers [27]. Overexpression of miR-30c and miR-544a has been reported to downregulate E-cadherin and upregulate vimentin that thereby stimulate EMT [86, 87]. Meanwhile, anti-miR-1246 and anti-miR-1290 can decrease the expression of CSCs markers and EMT markers in NSCLC [88]. MiR-221 and miR-222 exert their oncogenic abilities in part *via* upregulating the EMT-inducing gene ZEB2 through TRPS1 [89]. It suggests that inhibition of these miRNAs suppress the invasiveness of NSCLC cells.

Furthermore, a plenty of miRNAs have been shown to reduce sensitivity and acquired resistance to EGFR-TKI in lung cancer cells, including miR-23a. It is regulated by the TGF-β/Smad pathway and affects the EMT event through target E-cadherin. Because epithelial phenotype is less resistance to EGFR-TKIs, restoration of E-cadherin may increase the sensitivity of lung cancer cells to EGFR-TKIs. This study highlights that miR-23a may be useful as a new therapeutic target to overcome the EGFR-TKI resistance in NSCLC [90]. In addition to miR-23a, overexpression of miR-134 and miR-487b has reported to promote the EMT phenomenon and induce the drug resistance to gefitinib. Similarly, knockdown of these miRNAs can inhibit EMT and then reverse TGF-β1-induced resistance to gefitinib in NSCLC. Membrane-associated guanylate kinase, WW, and PDZ domain-containing protein 2 (MAGI2) is a direct target of miR-134 and miR-487b. Its suppression causes the loss of PTEN stability and PI3K/AKT activation. Kitamura et al. has reported that absence of PTEN-MAGI2 is related to acquire EGFR-TKI resistance *in vitro* [91].

## LNCRNAS-RELATED EMT IN NSCLC

### LncRNAs suppress tumor metastasis by reversing EMT

To date, a growing number of publications emphasize the role of lncRNAs play in tumor initiation, progression, and metastasis. This novel class of ncRNAs gives hope for a more profound understanding of lung cancer. Recent research has linked the dysregulation of lncRNAs with cell invasion in NSCLC [15]. BRAF-activated non-coding RNA (BANCR) is on chromosome 9 with the size of 693 bp. It has been found that the expression of BANCR was significantly downregulated at the later stages of tumor development, which is consist with lower overall survival time of patients with NSCLC. Most investigations agree on a major role for BANCR in enforcing EMT. Indeed, sun and colleagues reported that alteration of BANCR expression promoted cell migration and invasion *in vitro* [92]. MMP2 is a member of Matrix metalloproteases (MMPs). Upregulation of BANCR leads to a decrease of its protein level in NSCLC cells, which finally stimulating E-cadherin expression and reducing Vimentin expression. Taking this into account, BANCR possibly suppress the metastasis ability of NSLC cell partly through regulation of EMT, shedding new light on therapeutic approach [92].

### LncRNAs promote tumor metastasis by inducing EMT

Currently, lncRNAs is possible the key in the development of novel cancer treatment because targeting oncogenic lncRNAs is more direct to induce anticancer effect [16]. MALAT1 (also known as NEAT2), as the first identified lncRNA in lung cancer, is well studied in recent decades. This highly conserved lncRNA locates on 11q13, whose length is more than 8000 nt [93]. High level of MALAT1 has been reported to associate with lung cancer metastasis, which is crucially related to poor prognosis. In this study, alteration of E-cadherin and Vimentin also has been observed in the highly invasive subline of brain metastasis lung cancer cells. In conclusion, MALAT1 can promote brain metastasis *via* inducing EMT in NSCLC [94]. Besides, increasing evidence shows that downregulation of MALAT1 will alter the expression of EMT-associated genes, finally decreasing ZEB1, ZEB2 and Slug levels and increasing E-cadherin level [16].

HOTAIR belongs to lncRNA chain with 2158 nucleotide long, which is located on chromosome 12q13.13. Recent studies have reported that the level of HOTAIR expression is distinctly upregulated in tumor tissues, including breast cancer, liver cancer, ovarian cancer, gastric cancer, and NSCLC [95]. Alves et al. first showed that HOTAIR is required to tumor metastasis for its key role in different signaling mechanisms related to EMT [96]. HOTAIR selectively binds to polycomb repressive complex 2(PRC2) to regulate the chromatin methylation state. PRC2 is required for H3K27 trimethylation, which contains H3K27 histone methyl transferase EZH2, SUZ12 and EED [97]. Both EZH2 and SUZ12 have been identified to mediate the repression of gene E-cadherin by H3K27 trimethylation [98, 99]. These findings suggest that the activation of EMT is due, in part, to the interaction of HOTAIR with the PRC2 in lung cancer cells.

Although cigarette smoke is recognized as a strong risk factor for the development of lung cancer, the related molecular mechanisms remain unclear. In CSE-exposed HBE cells, MALAT1 affects EMT *via* binding to EZH2, and is negatively regulated by miR-217 [100]. Moreover, CSE has been demonstrated to induce secretion of IL-6, and then act on STAT3, which bind to HOTAIR promoter directly. Thus, the activity of HOTAIR finally contributes to EMT and CSCs [101]. These results provide a better understanding of the processes associated with NSCLC metastasis caused by cigarette smoke.

## CONCLUSIONS AND PERSPECTIVES

Despite great progress in early detection of NSCLC, almost 50% patients combine with developing distant metastases when they are first diagnosed [1]. Worse still, resistance to conventional therapeutics or advanced therapies presents an emerging challenge in the treatment of patients with NSCLC [6]. New biomarkers and druggable targets must be considered.

Currently, evidence is accumulating that shows a crucial role of EMT in tumor metastasis [26]. Some investigators have reported that losing expression of EMT markers such as E-cadherin and cytokeratin, and gaining expression of mesenchymal markers such as N-cadherin and vitmentin is significantly associated with advanced NSCLC stage as well as poor prognosis [102]. Furthermore, EMT-derived NSCLC cells acquire stem cell properties and exhibit resistance to chemotherapy, radiotherapy and target agents [53]. Molecules in the regulatory networks linking EMT are starting to emerge as potential molecular biomarkers and therapeutic targets for improving current cancer therapies and overcoming resistance. Non-coding RNAs has gained more attention in recent studies for its dramatic role in regulating EMT progress. Although without the ability of encoding proteins, they are emerging as central players in various cellular pathways, including EMT, through the targeting of a plenty of mRNAs [103].

NcRNAs are emerging as central players in the development of NSCLC *via* regulating the EMT progress (Table 1). MiRNAs is the small ncRNA chain, and gaining better understanding in the last decades. The functional properties make miRNAs appealing biomarkers and potential therapeutic targets in clinic. It is well known that targeting one miRNAs may affect the expression of various vital molecules simultaneously. For example, miR-200c can suppress the induction of EMT in part through targeting HFE, ZEB2 and USP25 respectively [66, 80]. However, in different lung cell lines, the same miRNAs seem to play an opposite role. Tumor-suppressive effect of miR-221 and miR-222 has been observed in H3255and HCC4006. In the contrary, both of these miRNAs promote invasiveness in H460 [89]. Furthermore, miR-134 seems to have a dual role in the development of lung cancer progression. It has been reported to promote the EMT phenomenon *via* targeting MAGI2 in A549 cells, whereas it also shows oncogenic ability in the same cell lines [91, 104]. The trait of these miRNAs may be a useful tool to predict whether they function as a tumor suppressor or not when efficient methods is developed.

**Table 1:** MicroRNAs related to EMT in NSCLC

MicroRNA	Role of microRNA	Target gene	Reference
miR-124	inhibit EMT	CDH2	[56]
miR-138	sensitize NSCLC cells to ADM	ZEB2	[77]
miR-32	inhibit EMT	TWIST1	[73]
miR-200 family			
miR-200c	inhibit EMT, radiosensitize A549 cells	ZEB2, SNAIL, N-cadherin, VEGF-VEGFR2 pathway	[60, 62–65]
miR-200b	inhibit EMT and diminish the erlotinib resistance of A549M cells	DLC1, HNRNPA3	[78, 80]
miR-452	inhibit EMT	PI3K/AKT pathway	[72]
miR-33a	inhibit EMT	TWIST1	[74]
miR-135a	inhibit EMT	KLF8	[57]
miR-205	inhibit EMT	ZEB1,Src	[79, 103]
miR-489	inhibit EMT	SUZ12	[67]
miR-129	inhibit EMT	MCRS1	[71]
let 7	inhibit EMT	HMGA2	[76]
let 7c	inhibit EMT and diminished the erlotinib resistance	unclear	[80]
miR-638	inhibit EMT	SOX2	[68]
miR-17	Inhibit EMT, diminish cisplatin-resistance	TGFβR2	[83]
miR-132	inhibit EMT	ZEB1	[66]
miR-193a-3p	inhibit EMT	ERBB4,S6K2	[59]
miR-193a-5p	inhibit EMT	PIK3R3,mTOR	[59]
miR-34a	inhibition of EMT, radiosensitize NSCLC cells	Notch-1	[82]
miR-149	inhibit EMT	FOXM1	[69]
miR-30a	inhibit EMT	Snial1,Vimentin	[75]
miR-148a	inhibit EMT	ROCK1	[58]
miR-1246&miR-1290	induce EMT	unclear	[86]
miR-221&miR-222	induce EMT	PTEN	[87]
miR-30c	induce EMT	E-cadherin, snail and vimentin	[84]
miR-134/487b/655	induce EMT, affect the drug resistance to gefitinib	MAGI2	[89]
miR-23a	induce EMT, promote resistant to gefitinib	E-cadherin	[88]
miR-544a	induce EMT	CDH2,vimentin	[85]

LncRNAs, another important component of ncRNAs, open new opportunities in understanding the role of EMT in metastases (Table 2). Recently, the competing endogenous RNA (ceRNA) hypothesis has described the lncRNA-miRNA-mRNA ceRNA network, where lncRNAs harbor miRNA response elements (MREs). Special lncRNAs could regulate the accumulation of miRNAs and in turn influencing the stability of protein complex [105]. For example, lncRNA unigene56159 inhibits the expression of Slug *via* directly binding to miR-140-5p, thereby promoting EMT in hepatocellular carcinoma (HCC) [106]. Similarly, lncRNA HULC/miR-200a-3p/ZEB1 axis facilitates HCC cells migration and invasion [107]. Also, in NSCLC, NEAT1 functions as a ceRNA for miR-377-3P. MiR-377-3p suppresses cancer progression through the activation of E2F3 pathway [108]. According to recent evidence, it is promising that lncRNAs regulate EMT *via* acting as ceRNAs in NSCLC cell lines. More effort is necessary to find the underlying function of lncRNAs.

**Table 2:** LncRNAs function as regulators of EMT during NSCLC metastasi

LncRNA	Roles of lncRNA	Target gene	Reference
BANCR	Inhibit EMT	MMP2	[90]
MALAT1	Promote EMT	E-cadherin, Vimentin, EZH2	[92, 98]
HOTAIR	Induce EMT	PRC2	[99]

In the prospective studies of EMT, circular RNA (circRNA), a potential class of ncRNAs, may capture the interest of researchers. Lately, abundant circRNAs have been demonstrated to exist in animal cells with regulatory potency. Contrary to miRNAs, circRNAs may partially act as post-transcriptional regulators [109]. Hundreds of cicrRNAs are regulated by specific factors during EMT [110]. Hence, it is with great anticipation that EMT-related circRNAs will been found in diseases, including NSCLC.

In summary, the inhibition of EMT could be a useful approach for inhibition cell invasiveness in NSCLC. And as such, the reversal of EMT, particularly through manipulating relevant ncRNAs, could also be useful for resensitization of NSCLC cells to conventional therapeutics, which would likely contribute significant improvements in treatment response.
